# Evaluation of the Clearview^® ^malaria pLDH malaria rapid diagnostic test in a non-endemic setting

**DOI:** 10.1186/1475-2875-10-284

**Published:** 2011-09-27

**Authors:** Sandrine Houzé, Véronique Hubert, Dorit Pessler Cohen, Baruch Rivetz, Jacques Le Bras

**Affiliations:** 1 Laboratory of Parasitology, Malaria National Reference Centre, AP-HP, Bichat Hospital, 46 rue Henri Huchard, 75018 Paris, France; 2 Orgenics Ltd, P.O.Box 360, Yavne 70650, Israel

## Abstract

**Background:**

Malaria Rapid Diagnostic Tests (RDTs) are widely used to diagnose malaria. The present study evaluated a new RDT, the Clearview^® ^Malaria pLDH test targeting the pan-*Plasmodium *antigen lactate dehydrogenase (pLDH).

**Methods:**

The Clearview^® ^Malaria pLDH test was evaluated on fresh samples obtained in returned international travellers using microscopy corrected by PCR as the reference method. Included samples were *Plasmodium falciparum *(139), *Plasmodium vivax *(22), *Plasmodium ovale *(20), *Plasmodium malariae *(7), and 102 negative.

**Results:**

Overall sensitivity for the detection of *Plasmodium spp *was 93.2%. For *P. falciparum*, the sensitivity was 98.6%; for *P. vivax*, *P. ovale *and *P. malariae*, overall sensitivities were 90.9%, 60.0% and 85.7% respectively. For *P. falciparum *and for *P. vivax*, the sensitivities increased to 100% at parasite densities above 100/μl. The specificity was 100%. The test was easily to perform and the result was stable for at least 1 hour.

**Conclusion:**

The Clearview^® ^Malaria pLDH was efficient for the diagnosis of malaria. The test was very sensitive for *P. falciparum *and *P. vivax *detection. The sensitivities for *P. ovale *and *P. malariae *were better than other RDTs

## Background

Malaria is a widespread and life-threatening disease caused by five species of *Plasmodium *in humans. Each year, 2, 200 imported malaria cases are reported to the French Malaria National Reference Center (FMNRC) among returned international travellers, and the real number of cases is estimated at 4, 000 [1]. Prompt diagnosis is essential for the treatment and outcome. Traditional standard diagnosis of malaria is based on microscopic examination of stained blood smears and it requires considerable training and experience. Most laboratories in non-endemic countries lack sufficient samples to enable building-up and maintenance of microscopic expertise [2]. Malaria rapid diagnostic tests (malaria RDTs) may be a complement to microscopic malaria diagnosis to non-expericenced laboratory staff in non-endemic setting. Malaria RDTs are immunochromatographic tests targeting specific antigens of one or more *Plasmodium *species. Among these antigens, the pLDH (*Plasmodium *lactate dehydrogenase) is a pan antigen, produced by all *Plasmodium *species parasites that infect humans: its detection allows the diagnosis of malaria cases whatever the *Plasmodium *species involved [3]. Malaria RDTs are available as strips or cassettes, and the two-band test display visible cherry-red to purple coloured control and test lines. By now, more than 60 RDT brands and over 200 different products have been developed. Of those, the World Health Organization (WHO) and Foundation for Innovative New Diagnostics (FIND) evaluated 70 from 34 manufacturers [4]. Of these products, only six are two-bands tests that detect pLDH. The Clearview^® ^malaria pLDH (Orgenics Ltd, Alere Diagnostics, Yavne, Israel) is a two-band RDT in a cassette format targeting the pLDH. In this study, their performance was evaluated with fresh blood samples of returned international travellers in a reference centre.

## Methods

### Study design

The Clearview Malaria pLDH (Orgenics) was evaluated on fresh blood samples received for analysis and obtained from international travellers suspected of malaria. Tests were carried out in the French Malaria National Reference Centre (FMNRC) in Bichat Hospital of Paris (France). The reference method was the microscopy performed on the samples. All discordant results between microscopy and the Clearview Malaria pLDH test were analysed by polymerase chain reaction (PCR) and test characteristics were recalculated according to the PCR-corrected results. The study design was in compliance with the STARD guidelines for presentation of diagnostic studies [5].

### Samples and materials

Samples are included between July and October 2010 from the collection of fresh venous EDTA-blood samples received in the laboratory for malaria analysis during the study in order to obtain 200 positive samples, and one hundred of negative samples. The patients were Europeans travellers returning from malaria-endemic countries and immigrants returning from visits to their native countries. Negative samples were obtained from febrile patients presenting at the outpatient clinic of Bichat Hospital and positive samples were obtained from patients diagnosed with malaria and sent by hospital laboratories to the FMNRC for complementary analysis in the scope of the national reference laboratory. Samples were considered as negative if no malaria parasite was observed by microscopy. The positive selected panel included samples at varying parasite densities with the four malaria species (*Plasmodium falciparum*, *Plasmodium vivax*, *Plasmodium ovale *and *Plasmodium malariae*) according to the proportion of each species observed in France. For *P. falciparum*, which is the most frequently retrieved species at FMNRC, a panel representing the different parasite densities was selected.

### Reference method

Microscopy, corrected by PCR, was used as the reference method. All samples were blindly analyzed by standard microscopy including species identification and determination of parasite density. After staining with Giemsa, thick and thin blood films were examined by an experienced microscopist in the laboratory. As recommended by the WHO, a slide was considered positive if at least one asexual form of parasite was detected in 100 microscopic fields in the thick blood film. Blood parasite density was determined by counting the number of parasites against between 200 and 1, 000 white blood cells (WBC) and assuming that each subject had 8, 000 white blood cells/μl of blood. The detection limits of microscopy in these conditions of reading were eight parasites per microliter. In case of the presence of *P. falciparum *gametocytes alone in the slide, the sample was not considered as positive. A species-specific PCR analysis as described by Snounou, was performed on all samples with non-*falciparum *species to confirm the species identification by microscopy [6].

### Test platform

Clearview^® ^malaria pLDH is a lateral-flow malaria RDT in a cassette format. Two lines are present, a control line which indicates whether the test is valid and a pLDH line indicates an infection with a *Plasmodium *spp. According to the manufacturer's instructions, Clearview^® ^malaria pLDH can detect *P. falciparum*, *P. vivax*, *P. ovale *and *P. malariae*. The kits used for the evaluation were stored at a dry place between 18°C and 25°C.

### Test procedure

Tests were performed according to the instructions of the manufacturer. Samples (5 μl) were loaded with a transfer pipette (Eppendorf, Le Pecq, France). In case of the control line did not appear, the result was interpreted as invalid and the test was repeated. Readings were performed 20 minutes after application of the sample and diluent. A second reading was performed 1 hour after application of the reagents. Results were scored as negative (no line visible), or positive (presence of the test line)(Table 1). If the test line was barely visible, the result was scored as doubtful. The band strength was visually compared to the intensity of the control line and scored as medium if the intensity of both the control line and the test line were equal, weak if the intensity of the control line was higher than the intensity of the test line and strong if the intensity of the control line was lower than the intensity of the test line.

**Table 1:** interpretation of the Clearview^® ^Malaria pLDH results according to the microscopy

Microscopy	**Clearview**^**® **^**Malaria pLDH**	Classification of the test
Negative	Negative	True négative

Positive for malaria (P.f. or P.v. or P. o. or P. m.)*	Positive or doubtful	True positive

Positive for malaria (P.f. or P.v. or P. o. or P. m.)*	Negative	False négative

Negative	Positive or doubtful	False positive

* P.f.: Plasmodium falciparum, P.v.: P. vivax, P.o.: P. ovale, P.m.: P. malariae.

### Analysis of discordant results

In case of discordant results between RDT and microscopy (false-negative and false-positive RDT results)(Table 1), a species specific PCR was performed on both *P. falciparum *positive or negative samples [6].

### Statistical analysis

Sensitivity and specificity were calculated for overall *Plasmodium *species and for each species with 95% confidence intervals (C I). The interpretation of the test result is shown in Table 1. The test result was considered as positive with presence of the both control line and pLDH line, and the test was considered as negative in case of the presence of the control line alone.

## Results

### Samples collections

The final panel consisted of 292 samples including infections with *P. falciparum *(n = 139), *P. vivax *(n = 22), *P. ovale *(n = 20), *P. malariae *(n = 7), *P. falciparum *and *P. ovale *(n = 1), *P. falciparum *and *P. malariae *(n = 1), and *Plasmodium *negative samples (n = 102) (Table 2). No sample with pure gametocytaemia was included. These samples were obtained in 292 patients with a male:female ratio of 1.6. The median age was 35 years (range 2-75). Travellers returned from Africa (n = 250), Latin America and South America and Caribbean (n = 30) and Asia (n = 12). Most *P. falciparum *infections had been acquired in sub-Saharan Africa (Table 2).

**Table 2:** species distribution, parasite densities and geographical origin of the samples included for the Clearview^® ^Malaria pLDH evaluation.

Geography	Species identification by microscopy corrected by PCR				
	
	*P. falciparum* (alone or in association)	*P. vivax*	*P. ovale*	*P. malariae*	No parasites
Africa	139		20	7	84
Asia		4			8
Latin and south America	2	18			10

Parasite density [median; p/μl] (range)	123, 762 (32-2, 250, 000)	4, 594 (8-35, 550)	4, 596 (225-21, 150)	2, 083 (135-8, 550)	

### Quality control of microscopy results

All PCR performed on samples with discordant results between RDT and microscopy, confirmed the microscopy results.

### Tests characteristics

No invalid test results were observed. Table 3 shows the test characteristics according to the species identification and parasite density. The overall sensitivity of the test and 95% CI for the diagnosis of malaria was 93.2% [88.3-96.2] (177/190). For *P. falciparum*, the overall sensitivity was 98.6% [94.9-99.6] (137/139). The sensitivities for non-*falciparum *species were 90.9% [72.2-97.4] for *P. vivax *(20/22), 85.7% [48.7-97.4] for *P. malariae *(6/7), and 60% [38.7-78.1] for *P. ovale *(12/20).

**Table 3:** specificity and sensitivity of Clearview^® ^Malaria pLDH for the detection of all *Plasmodium *species.

Microscopic examination (confirmed by PCR)	Clearview Malaria pLDH		Specificity (95 CI%)	Sensitivity (95 CI%)
	positive	negative		
Negative		102	100% (96.4-100)	

Positive *P. falciparum*	137	2		98.6% (94.9-99.6)

Positive *P. vivax*	20	2		90.9% (72.2-97.5)

Positive *P. ovale*	12	8		60.0% (38.7-78.1)

Positive *P. malariae*	6	1		85.7% (48.7-97.4)

*P. falciparum and P. malariae*	1			ND*

*P. falciparum *and *P. ovale*	1			ND*

* ND: not determined

Parasite densities of false negative samples were comprised between 225 and 21, 150 p/μl for *P. ovale*, equal to 40 or 540 p/μl for *P. vivax*, equal to 900 p/μl for *P. malariae*, and equal to 32 or 64 p/μl for *P. falciparum*. For both *P. falciparum *and *P. vivax*, the sensitivity increased to 100% at parasite densities above 100 p/μl. None of the *Plasmodium *negative samples was scored as positive, so the specificity was 100% [95.5-100].

### Line intensity reading

For the majority of samples, the positive test line was read as strong and medium. The line intensity reading was weak for 11 samples: eight *P. falciparum *infected samples with parasite densities comprised between 112 p/μl and 1350 p/μl; two samples infected by *P. vivax *with parasite densities equal to 8 p/μl and 900 p/μl and one sample infected by *P. ovale *with a parasite density equal to 9900 p/μl

### Ease of use and stability of the result

The Clearview pLDH test was easily to perform: no difficulty had been reported to transfer the blood or the reagent. The clearing of the test strip was good and the test lines were very easy visualized. The result is stable for a long time, more than one hour: no positive sample appeared negative after one hour and inversely, and no change in the band intensity was observed. The test instructions in the package insert were scored as clear and the use of pictures was well appreciated.

## Discussion

In this prospective study, the performance of the Clearview pLDH test was evaluated on samples obtained in returned travellers suspected of malaria. Overall sensitivity for the detection of *Plasmodium *was 93.2%. For the detection of *P. falciparum*, the sensitivity was 98.6%, reaching 100% in samples with parasite densities above 100/μl. Overall sensitivities for *P. vivax*, *P. malariae *and *P. ovale *were 90.9%, 85.7% and 60.0% respectively. No positive result occurred among the *Plasmodium *negative samples.

This study found similar sensitivity of this RDT for the detection of *P. falciparum *than a PfHRP2-based RDT [7]. The advantage of the pLDH test for the diagnosis of *P. falciparum *is the absence of genetic variation in the pLDH gene [8] in contrast with PfHRP2: samples of *P. falciparum *were reported without PfHRP 2 gene in Amazon [9]. In comparison with RDTs targeting pLDH included in the WHO/FIND evaluation, the sensitivity of Clearview^® ^pLDH is higher for both *P. falciparum *and *P. vivax *detection. In the WHO evaluation, for *P. falciparum*, only two tests of the 27 tested products had a sensitivity higher than 95% at low parasite density (200 parasites/μl): these tests targeted PfHRP2 [4].

For *P. vivax *samples at low parasite densities, of the 20 tested, only four products had a sensitivity higher than 90%.

Similar sensitivity (91.0%) for the detection of *P. vivax *was reported in a study in Myanmar with the Malaria pLDH test (CareStart^® ^brand) [10]. Ashton *et al *reported similar sensitivities with three different tests in the range of 82.5% to 85.0% observed in Ethiopia [11]. In non-endemic countries, studies evaluating other RDTs detecting pan-pLDH showed sensitivities of 66.0%, 77.6% or 87.5% for *P. vivax *detection [12-14].

Studies evaluated the sensitivities for *P. malariae *and *P. ovale *are few and in all cases, sensitivities for detection of these species were poor. Few RDT tests had sensitivity for the detection of *P. malariae *higher than 80%, like the Clearview^® ^test. Previous studies performed in non endemic setting, reported sensitivities for detection of *P. malariae *with a pLDH based RDT test between 30.4% [13] and 45.2% [14].

The Clearview^® ^pLDH test is better than more of the RDT tests for the detection of *P. ovale *despite a false-negative result with a high parasitaemia (21, 150 p/μl) confirmed after repeating the RDT. A lack of pLDH production by the involved parasite could explain this result as demonstrated for PfHRP2 in *P. falciparum *[15]. Genotype analysis could be performing to confirm this hypothesis. For this species diagnosis, recent studies reported 5.5% for the Palutop +4 [12] or 18.4% for the CareStart^® ^Malaria Pf/pan [13]. The best test seemed to be the SD FK60 Malaria Ag Pf/Pan test with a sensitivity of 76.3% [14].

The specificity of the test is excellent: no false positive result was observed. Especially, the advantage of the pLDH detection is the clearance of the protein after anti-malarial treatment, so no false positive result was observed in treated patient like with PfHRP2 RDT based test [16].

This evaluation of the RDT in a reference setting give data before using in a hospital setting in a developed country. Evaluation in the field would be necessary to confirm these performances in other circumstances.

The test was easily to perform with good clearing of the background. The line intensity was stable in time, more than one hour. The small volume of blood used to perform the test is an advantage especially when the test is performed with capillary blood from children regarding to the difficulties in collecting blood from young children. Most of RDT needs at least 10 μl of blood to perform the test.

## Conclusions

The Clearview^® ^pLDH test is very specific and sensitive test for all *Plasmodium *species responsible of imported malaria in France. The sensitivity for the detection of *P. ovale *is medium but better than other RDT tests available in France. The test is easily to perform and result could be re-read a long time after it had been performed, which is very important to control results, for example those obtained during night-duty.

## Competing interests

SH, JLB and VH declare that they have no competing interests. BR and DPC are members of the Alere company.

## Authors' contributions

SH, BR and DPC participated in the design of the study. SH carried out the immunoassays, performed the statistical analysis and drafted the manuscript. VH performed the PCR assays. JLB and BR helped to draft the manuscript. All authors read and approved the final manuscript.
